# Role of the mechanical microenvironment in cancer development and progression

**DOI:** 10.20892/j.issn.2095-3941.2019.0437

**Published:** 2020-05-15

**Authors:** Qiuping Liu, Qing Luo, Yang Ju, Guanbin Song

**Affiliations:** ^1^Key Laboratory of Biorheological Science and Technology, Ministry of Education, College of Bioengineering, Chongqing University, Chongqing 400030, China; ^2^Department of Mechanical Science and Engineering, Nagoya University, Nagoya 464-8603, Japan

**Keywords:** Cancer stem cell, cell metabolism, mechanical force, tumor progression

## Abstract

Cross-talk between tumor cells and mechanical stress in the tumor microenvironment has been shown to be involved in carcinogenesis. High mechanical stress in tumors can alter the metabolism and behaviors of cancer cells and cause cancer cells to attain cancer stem-like cell properties, thus driving tumor progression and promoting metastasis. The mechanical signal is converted into a biochemical signal that activates tumorigenic signaling pathways through mechanotransduction. Herein, we describe the physical changes occurring during reprogramming of cancer cell metabolism, which regulate cancer stem cell functions and promote tumor progression and aggression. Furthermore, we highlight emerging therapeutic strategies targeting mechanotransduction signaling pathways.

## Introduction

Despite many efforts to cure cancer, it remains a leading cause of death worldwide. Cancer develops within a complex tissue microenvironment that promotes epigenetic reprogramming and modification of the tumor phenotype^1^. Moreover, an aberrant microenvironment plays important roles in the growth, invasion, and metastasis of tumor cells. The unique interplay among various aspects of tumor cells and the microenvironment can yield molecular targets for tumor treatment. Fortunately, the role of the tumor microenvironment, and its cellular and molecular composition, along with chemical and physical factors involved in tumor development, have received increasing research interest. Several studies have confirmed the contributions of the cellular and molecular composition of the tumor microenvironment to cancer development and progress^2^. However, the effects of physical stimulation remain to be fully clarified.

Many studies have focused on genetic and biochemical factors as the causes of malignant tumors. However, physical factors have been generally ignored. Tumor cells are usually limited to a specific microenvironment, such as the extracellular matrix (ECM), and micro-environmental changes can affect tumor cell behavior. Thus, the mechanical properties of the microenvironment also play critical roles in cancer development, relapse, and metastasis. Tumor growth and development are accompanied by changes in mechanical factors in the tumor microenvironment, such as tumor solid stress, matrix stiffness, and strengthening interstitial fluid flow induced by unmitigated increases in interstitial hydraulic pressure^3^. The critical role of mechanics in cancer progression has been confirmed in the past decade^4–6^. In this review, to facilitate understanding of how mechanical forces influence cancer development and progression, we discuss the roles of mechanical forces in promoting the metabolic reprogramming of cancer cells and stemness maintenance of cancer stem cells (CSCs) through mechanotransduction.

## Tumor microenvironment

### Components of the tumor microenvironment

The tumor microenvironment (TME) is a complex mixture of tumor cells, stromal cells, immune cells, carcinoma associated fibroblasts, and non-cellular components within the ECM^7^. Inappropriate disruption of the TME in cancer contributes to the malignant characteristics of tumor cells and cancer progression^1^. Studies have suggested that the TME plays a pivotal role in tumor initiation, progression, metastasis, and therapeutic efficacy. Carcinoma associated fibroblasts compose the largest proportion of the stromal cells and mainly have promoting roles in metastasis initiation^8^. Immune cells are present and interact with the tumor cells *via* direct contact or through chemokine and cytokine signaling, thus shaping the tumor’s behavior and response to therapy. For example, gastric cancer cells inhibit natural killer cell proliferation and induce apoptosis *via* prostaglandin E2^9^. In addition, the TEM significantly influences therapeutic responses and clinical outcomes^10^, and the immunoScore signature is used as a prognostic and predictive tool in cancer^11,12^. Jiang et al.^13^ have built a model to improve the overall prediction of outcomes for patients with gastric cancer according to the tumor immune microenvironment and chemosensitivity signature. Moreover, the plastic properties of mesenchymal stromal cells triggered by the TME have been found to induce malignant neoplastic tissue formation, maintenance, and chemoresistance, as well as tumor growth^14^. Bone marrow stromal cells have also been reported to mediate chemoresistance in acute myeloid leukemia *via* Notch signaling^15^. Therefore, the components of the TEM are crucial to tumor development.

### Mechanical forces in the TME

The microenvironment of tumor tissues is different from that of normal tissues, as reflected mainly in the abnormal structure and function of blood vessels and lymphatic vessels, high stroma pressure, and a dense interstitial matrix^16^. Recent studies have emphasized that, in addition to biochemical signals from the microenvironment, physical signals can significantly change cell behavior, such as proliferation and metastatic potential, beyond the characteristics of cancer stem cells. The physical signals in tumors comprise mainly 3 aspects: increasing matrix stiffness, solid stress, and interstitial fluid pressure^17,18^. These forces do not operate independently within the tumor but instead interact during carcinoma development and progression.

ECM remodeling and stiffening are characteristics of solid tumors, and tissue stiffness has been exploited to detect a variety of human cancer types^19^. The stiffness of breast cancer tissue is approximately 10 times that of normal breast tissues^20^. Chronic liver diseases leading to hepatic carcinoma are associated with ECM over-production, and the stiffness of liver cancer tissue is approximately 10 times that of normal liver tissue^21^. ECM stiffening in tumors is caused by reorganization of the stroma by excess activity of ECM proteins and enzymes that covalently cross-link collagen fibers and other ECM components^6,22^. For example, hepatic stellate cells are activated in response to liver damage, thus resulting in extensive accumulation of ECM and leading to the development of hepatic fibrosis, or even hepatic cirrhosis and hepatic carcinoma^23,24^. Moreover, overexpression of lysyl oxidase in cancers has been attributed to an increase in tissue stiffness by cross-linking collagen fibers and other ECM components. As a biomechanical property of solid tumors, increased tissue stiffness has been widely and actively studied, and is considered to be involved in regulating several tumor characteristics, including growth, metabolism, invasion, and metastasis^2,18,25^. However, how stiffening of the ECM drives tumor progression remains to be determined.

Growth-induced solid stress accumulates within tumors during tumorigenesis and the rapid proliferation of tumor cells^18^. Solid tumors grow under compressive stress, which corresponds to mechanical loads of 35–142 mm Hg for human tumors^26,27^. Solid stress is contained in and transmitted by ECM and cellular elements^28^, and it can affect the growth of cancer cells both directly, by compressing cancer cells, and indirectly, by compressing surrounding blood and lymphatic vessels^29^.

Fluid stresses include microvascular and interstitial fluid pressure as well as the shear stress exerted by blood and lymphatic flow on the vessel wall, and by interstitial flow on cancer and stromal cells and ECM^18^. High interstitial fluid pressure is another characteristic of solid tumors, which results from solid stress and accumulation of fluid in the interstitial space^30,31^. The interstitial fluid pressure can direct tumor cell migration through autocrine CCR7 signaling^32^. Hyler et al.^33^ have suggested that even a low level of continual fluid shear stress significantly and differentially affects adherent epithelial ovarian cancer cells in various stages of progression. As described above, interstitial fluid pressure within the TME can direct cell movement and promote tumorigenesis.

### Abnormal metabolic microenvironment in tumors

Metabolic reprogramming, a defining feature of almost all cancers, is a robust hallmark in addition to the 6 recognized hallmarks of cancer^34,35^. In addition, studies have proposed that cancer is a metabolic disease, thus causing a gradual shift in the view of cancer as a genetic disease^36,37^. The metabolic environment in solid tumors is characterized by hypoxia and acidity. These are important determinants of tumor cell growth and metabolism, and tumor resistance to radiation therapy, chemotherapy, and other therapies^38^. The main cause of low pH in the TME is H^+^ ions from lactic acid and carbonic acid, as a result of anaerobic glycolysis and the conversion of CO_2_ and H_2_O *via* carbonic anhydrase, respectively^38^. The formation of hypoxic areas in tumors is mainly caused by the distribution of the vascular network and uneven cell proliferation. The TEM usually lacks nutrients because of the elevated rates of nutrient consumption by tumors and inadequacies in the tumor vascular supply. Consequently, tumor cells must reprogram their metabolism to adapt to hypoxic, acidic, and low-nutrient conditions^39^.

In recent years, advances in cancer research have enhanced understanding of metabolism and its heterogeneity in cancer cells. In cancer, elevated glucose uptake and high glycolytic rates, as a source of adenosine triphosphate (the Warburg effect), are major metabolic characteristics of tumors. Thus, targeting the Warburg effect has been suggested as a “metabolic therapy” approach for the treatment of cancer^40^. However, advances in recent years have suggested that the metabolism in tumors not only conforms to the Warburg effect but also is heterogeneous. Indeed, studies have revealed a dual capacity of tumor cells for glycolytic and oxidative phosphorylation metabolism^41–43^. Metabolic plasticity of cancer cells helps them adapt to the TEM and promotes cancer development. Xu et al.^44^ have suggested that increased glucose metabolism, induced by high levels of fibroblast growth factor receptor 4 (FGFR4), can lead to chemoresistance in breast cancer. Shakery et al.^45^ have found that beta-hydroxybutyrate treatment decreases glycolysis and increases oxidative phosphorylation, thus fueling the proliferation, migration, and stemness of 5FU treated SW480 colon cancer cells. Therefore, metabolic reprogramming supports cancer cell stemness and bioenergy-consuming behaviors, such as proliferation, survival, migration, invasion, and chemoresistance^46^.

Tumor niche aberrant mechanical forces and tumor cell metabolic reprogramming have been reported to be 2 fundamental mediators of tumor progression, and recently a mechanistic interconnection between them has been established. Bertero et al.^47^ have elucidated that ECM stiffening induces a metabolic switch in both cancer and stromal cells. Furthermore, our recent study has shown that a stiffer matrix promotes glycolysis in HCC cells, thus allowing them to meet their energy needs for migration^48^. Therefore, the aberrant mechanical forces in the TME around an expanding tumor modulate cancer cell metabolism, thus supporting the metabolic requirements for tumor progression. However, little is known about the correlation between the mechanical force in TME and tumor metabolism, and further studies are needed to explore their effects in the occurrence and development of tumors.

## Intracellular signaling events in response to microenvironmental mechanics

**Figure 1:**
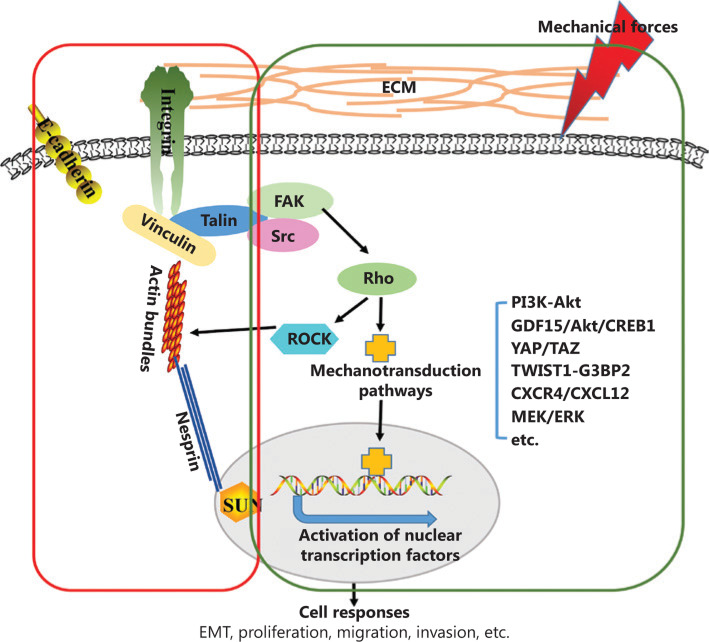
Schematic presentation of biomechanical force transmission from the extracellular environment into cancer cells. Changes in the mechanical properties of the TME transmit to cancer cells through 2 distinct pathways: the physical nuclear-cytoskeletal connection and biochemical signaling. Mechanical stress transmission occurs through physical nuclear-cytoskeletal connections involving integrins, F-actin, nesprin, and SUN proteins (red box). Moreover, integrin clustering and focal adhesion assembly, driven by biomechanical forces, activate biochemical signaling pathways such as PI3K-Akt, GDF15/Akt/CREB1, YAP/TAZ, TWIST1-G3BP2, CXCR4/CXCL12, and MEK/ERK (green box).

Biomechanical forces from the extracellular environment can be transduced or converted into intracellular signals, in a process referred to as mechanotransduction. This complex process involves a multitude of signaling molecules and events, operating both sequentially and in parallel^49^. Mechanosensitive molecules at the cell surface, such as integrins and cadherins at the adherens junctions, receptor tyrosine kinases, and ion channels, primarily sense physical signals^50^. Integrins are a widely studied family of mechanosensors^51^, which are key components of focal adhesion complexes. Integrins are transmembrane proteins that bind various ECM proteins and are involved in sensing the extracellular environment. As suggested by Yu et al.^17^, “integrins can mediate the sensing of mechanical properties of the ECM and transduce these signals downstream to focal adhesion kinase (FAK), leading to the stabilization of focal adhesions, and activation of downstream intracellular signaling cascades”. E-cadherin is another major mechanosensor involved in the sensing and transmission of force^7^. Integrin and cadherin complexes act as cellular mechanosensors and mechanotransducers at cell-ECM or cell-cell junctions, respectively^52^. To date, only a few of these mechanically sensitive molecules have been discovered, and future studies are needed to discover more mechanosensors and verify their roles in tumor development. After being sensed by mechanosensors, biomechanical signals can be transduced downstream to FAK and the nucleus through 2 pathways (as shown in **Figure 1**): biochemical mechanotransduction and direct transduction to the nucleus by physical anchoring of the cytoskeleton and nuclear lamina^53^. These 2 mechanotransduction pathways do not exist independently; in fact, they interact mutually and affect the characteristics of cancer cells, such as cell proliferation, adhesion, cytoskeletal remodeling, and migration^54–56^.

A variety of intracellular signaling pathways can be activated by mechanical signals, and the activation of intracellular signaling cascades affects the biological behavior of cancer cells (**Table 1**). Matrix stiffness potently regulates cellular behavior through various pathways. For example, matrix stiffness can drive epithelial-mesenchymal transition (EMT) and tumor metastasis through the TWIST1-G3BP2 mechanotransduction pathway^60^. Recently, Kalli et al.^66^ have reported that solid stress induces the migration of pancreatic cancer cells, in a process mediated by GDF15 through Akt pathway activation. In addition, transient receptor potential vanilloid 4 (TRPV4), which is sensitive to a wide variety of chemical and physical stimuli, is likely to mediate EMT, as induced by TGFβ1 and matrix stiffness^69^. Dupont et al.^70^ have demonstrated that yes-associated protein/transcriptional coactivator with PDZ-binding motif (YAP/TAZ) activity is regulated by ECM rigidity and cell shape. Moreover, YAP/TAZ mediate cellular mechanoresponses. Beyond matrix stiffness, other mechanical forces have been studied. Shah et al.^63^ have reported that interstitial fluid flow increases the invasion of hepatocellular carcinoma cell *via* CXCR4/CXCL12 and MEK/ERK signaling. In addition, a recent study has reported that hydrodynamic shear stress promotes EMT in human breast tumor cells through downregulation of ERK and GSK3β activity^65^. These studies suggest that biomechanical forces from the extracellular environment are transmitted into the intracellular environment and transformed into biochemical signals, thereby regulating the behavior of cancer cells.

Biomechanical signals can also be transmitted from the ECM to the internal cytoskeleton and transduced to the nucleus through physical nuclear-cytoskeletal connections (**Figure 1**). Complexes of nesprins and SUN-domain (Sad1/UNC-84) proteins, called linkers of nucleoskeleton and cytoskeleton (LINC) complexes, bind the cytoskeleton to the nucleus. This link between the cytoskeleton and the nucleus transmits mechanical signals that regulate nuclear position and cell behavior^71^. Inhibitors targeting mechanotransduction pathways (reviewed in^72^) have shown significant therapeutic effects in both preclinical models and clinical trials, thereby indicating the potential of targeting mechanotransduction in cancer therapies.

## Microenvironmental mechanics affects cancer progression

Mechanical imbalance is a major feature of malignant tumor tissue that increases the possibility of an imbalance in mechanical homeostasis becoming a precursor for tumorigenesis and progression^73^. In fact, matrix stiffening is associated with a variety of diseases, such as fibrosis or cirrhosis of tissues, thus increasing the risk of malignant tumors. For example, the continued development of liver fibrosis and liver cirrhosis leads to uncontrollable nodular hyperplasia in later stages and further develops into scirrhous hepatocellular carcinoma^74,75^. Angiogenesis, an essential hallmark of solid tumors, plays important roles in tumor growth and hematogenous metastasis^76,77^. As reviewed by Zanotelli et al.^78^, aberrant tumor angiogenesis is promoted by alterations in ECM mechanics in the TME. In addition, important biophysical parameters such as abnormal cytoskeletal or matrix mechanics are associated with many cancer hallmarks, including unlimited replicative potential, apoptotic evasion, and tissue invasion and metastasis^79^. Along with changes in the biomechanical characteristics of TME, an advantageous ‘niche’ is created that allows cancer cells/CSCs to turn on different mechanosensory pathways, and adjust their behaviors and metabolism.

### Mechanical forces influence cancer cell behavior

The physical interaction between cells and their ECM has been shown to affect many cellular behaviors associated with cancer progression through the regulation of master developmental pathways, such as Notch, Wnt, and Hedgehog^80^. As reported by Tse et al.^81^, compressive stress accumulates during tumor growth and makes cancer cells invasive. Studies by Helmlinger et al.^82^ and Delarue et al.^68^ have also suggested that compressive stress inhibits proliferation in tumor spheroids. McKenzie et al.^83^ have reported that ECM stiffness regulates ovarian cancer cell morphology, migration, and spheroid disaggregation. Moreover, increasing 3D rigidity has been demonstrated to promote proliferation and spheroid development of liver cells^84^. Solid stress in tumors, as experimentally mimicked by compression, has also been shown to alter the adhesion and migration of cancer cells^85^. In addition, a recent study has reported that increased TME stiffness stimulates the secretion of activin A (a strong pro-metastatic cytokine in cancer associated fibroblasts), and stromally secreted activin A induces ligand-dependent CRC epithelial cell migration and EMT^86^. These results indicate that mechanical forces in the TME promote aggressive behaviors of cancer cells, including proliferation, migration, invasion, and spheroid development.

### Microenvironmental mechanics affects cancer stem cells

Increasing evidence suggests that CSCs have many of the features essential to tumor initiation, invasion, and recurrence^87^. Recent studies have found that CSCs play crucial roles in liver cancer development, radio-chemotherapy resistance, recurrence, and metastasis^88–90^. CSCs, a subset of tumor cells, are also subject to mechanical force within the TME. Chen and Kumar have reviewed studies on CSC functions regulated by biophysical signals in the TME, including interstitial pressure and ECM stiffness^87^. The matrix stiffness of cancer tissue increases significantly from the center outward^21^, with increased expression of ECM components such as type I collagen and laminin. CSCs with high clonal expansion, invasiveness, and metastatic ability are mainly concentrated in the invasion frontier area of cancer tissue^91,92^. Notably, the microenvironment of this region is relatively more suitable for maintaining the stemness, invasiveness, and metastatic ability of CSCs. ECM components have been demonstrated not to be the key factors regulating the stemness, proliferation, and metastasis of CSCs; therefore, intratumor mechanical heterogeneity may be the cause. Further investigation of the biophysical regulation of CSCs may provide a promising approach to reveal new CSC specific targets for pharmacological intervention. In-depth study of the biophysical regulation of CSCs may help reveal the roles of mechanical factors in promoting the occurrence and development of cancer.

Although many researchers have conducted excellent work in cancer stem cell research, little is known about the origin of CSCs. Some evidence suggests that chemical factors may be involved in regulating the origin of cancer stem cells. For example, hypoxia enhances the generation of progenitor cell-induced pluripotent stem cells^93,94^ and contributes to maintenance of glioma stem-like cells^95^. Small molecules have also been reported to target the self-renewal, expansion, differentiation, and survival of endogenous stem cells^96,97^. In addition, mechanical factors have been found to play an important role in maintaining the stemness of CSCs. Mechanical factors (such as matrix mechanical properties) not only induce cancer cells to show characteristics of malignant transformation but also promote the expression of stem cell markers^98,99^. Recently, hydrodynamic shear stress has been demonstrated to promote the conversion of circulating tumor cells to distinct cancer stem-like cells in the blood circulation^47^. Our previous study has also demonstrated that a soft matrix increases the stemness of HCC cells^100^. Tumor tissues with impaired mechanical function are often accompanied by increased hypoxia^33^. Pang et al.^101^ have found that the combination of increased stiffness and decreased oxygen tension in the TME increases the expression of CSC markers in invasive breast cancer cells. Hence, targeting the mechanical forces in the CSC niche may provide a new approach for suppressing the TME-driven activation of CSCs.

### Microenvironmental mechanics and metabolic dysfunction

The metabolism of cancer cells is reprogrammed to preferentially use glycolysis rather than oxidative phosphorylation to obtain energy (Warburg effect). ECM stiffening and tumor cell metabolic reprogramming are 2 important characteristics during tumor progression that serve as important regulatory factors promoting cancer occurrence and development. Cancer cells have greater metabolic plasticity that allows them to better adapt to the changing TME. Pickup et al.^102^ have suggested that the mechanical features of the ECM may profoundly regulate many classic and emerging cancer hallmarks, including cellular metabolism. Transformation of mechanical signals into tumor-induced biochemical signals activates signaling pathways that regulate cancer cell metabolism to meet the energy requirements in malignancy. Therefore, mechanical stimuli from the TME may provide crucial molecular signals that guide metabolic reprogramming in tumor cells to favor aggressive behaviors^47^.

Mechanical stimuli in the TME have been suggested to activate signaling pathways that promote the aggressive behavior of cancer cells^73^. Interestingly, many of these signaling pathways regulate the metabolism of cancer cells and their malignant behaviors. The biophysical properties of the ECM regulate malignant transformation and tumor metastasis through the PI3K/AKT signaling pathway^2^, which is central in the regulation of glucose uptake and utilization^103,104^. Activation of the PI3K/AKT signaling pathway renders cells dependent on high levels of glucose flux. Thus, the regulation of matrix stiffness by the PI3K/AKT signaling pathway may affect cell metabolism, thus regulating cell proliferation and survival, and affecting tumor growth and metastasis. In addition, the YAP/TAZ-dependent mechanotransduction cascade is crucial to metabolic reprogramming initiated by ECM stiffness^28^. Recently, Bertero et al.^105^ have linked mechanical stimuli (ECM stiffening) to metabolic reprogramming through YAP/TAZ-dependent glutamate/aspartate cross-talk in the TME. In this study, ECM stiffening was demonstrated to reprogram cell metabolism, including increasing glycolysis and glutamine metabolism^105^. The activation of subcellular AMPK, a metabolic energy sensor, in MDA-MB-231 and MCF-10A has been found to be induced by fluid flow shear stress^106^. In another study, AMPK has been found to be activated in response to force applied to E-cadherin; moreover, force-induced AMPK increases glucose uptake and ATP levels in MCF10A^107^. These findings underscore the metabolic responses of cancer cells to mechanical stimuli in the tumor niche through a variety of signaling pathways. However, other pathways are likely to link mechanical stimuli to metabolism, and further studies are needed to fully investigate their complex relationships.

## Conclusions

The biomechanical changes in the TME can modify the behavior and metabolism of tumor cells, along with the properties of CSCs, thus promoting the development of cancer. Increased ECM stiffness, solid stress, and fluid stress within tumors are characteristics of cancer progression and activate signaling pathways critical for proliferation, survival, migration, invasion, and metastasis. Some of these mechanical force-activated signaling pathways in tumors also promote metabolic reprogramming. Furthermore, the biomechanical properties of the TME regulate properties of CSCs by modulating stemness-maintaining pathways through mechanotransduction. Tissue mechanosignaling activates signaling networks that simultaneously promote metabolic reprogramming and the maintenance of CSC characteristics. Altered biomechanical properties and metabolic reprogramming of tumor tissue and CSCs are critical drivers of cancer aggressiveness. Therefore, further studies on metabolic reprogramming and CSCs in the mechanical microenvironment, as well as their interconnected feedback mechanism, are important.

Co-existing biochemical and biomechanical signals in the TME cooperatively drive tumor progression. These signals include hypoxia and pH gradients; gradients of soluble signals and ions; and physical forces caused by modification of the concentration, organization, and stiffness of the ECM^108^. Cells can regulate directional responses to multiple signals through the same cell surface receptors or signaling pathways. Together, the mechanical forces, mechanoresponsive elements, biochemical signals, and cross-talk with intracellular signaling pathways regulate diverse cellular behaviors, cell metabolism, and the maintenance of CSC properties. However, many questions regarding the link between the TME and tumor progression remain to be answered. Understanding how cancer cells integrate multiple directional signals in the development and progression of tumors is critical to identify novel anticancer therapeutic targets.

## Figures and Tables

**Table 1:** Mechanical signal-transducing pathways in cancer

Physical signals	Mechanical signals	Stress parameters	Target molecules/pathways	Effectiveness of mechanical signals	References
Matrix stiffness	ECM rigidity	8.6–55 kPa	FAK/phosphopaxillin/vinculin pathway	Induce migration of mouse embryonic fibroblasts	^57^
	Matrix rigidity	1023, 7307, and 22,692 Pa	ROCK signaling	Enhance the invasive migration of cancer cells	^58^
	Matrix stiffness	1, 4, and 25 kPa	E-cadherin/β-catenin/YAP/TAZ	Induce EMT and promote chemoresistance in pancreatic cancer cells	^59^
	Matrix stiffness	150 and 5700 Pa	TWIST1-G3BP2	Drive EMT and tumor metastasis of breast tumors	^60^
	Microenvironmental stiffness	0.08–119 kPa	EGFR signaling	Enhance glioma cell proliferation	^61^
Shear stress	Fluid shear	2 dyne/cm^2^	IGF-2 and VEGF signaling pathways	Promote chondrosarcoma cell invasion	^62^
	Interstitial flow	0.2 µm/s	CCR7 signaling	Direct tumor cell migration	^32^
	Interstitial fluid flow	0.05–0.1 µm/s	CXCR4/CXCL12 and MEK/ERK	Increase invasion of hepatocellular carcinoma cell	^63^
	Low shear stress	1.8 dyne/cm^2^	FAK/Src and ROCK/p-MLC pathways	Induce breast cancer cell motility and adhesion	^64^
	Fluid shear stress	2.25–20 dyne/cm^2^	ERK-GSK3β	Promote EMT	^65^
Compressive stress	Solid stress	4.0 mm Hg	GDF15/Akt/CREB1 pathway	Induce migration of pancreatic cancer cells	^66^
	Compressive strains	1% strain	ERK1/2-RUNX2	Promote drug resistance of tumor cells	^67^
	Compressive stress	10 kPa	p27^Kip1^	Inhibit proliferation in tumor spheroids	^68^
